# Between work and care: a comparative study of the trajectories of women from different social classes in Cuba and Argentina

**DOI:** 10.3389/fsoc.2026.1756504

**Published:** 2026-04-22

**Authors:** Leticia Muñiz Terra, Dayma Echevarría León, Yenisei Bombino Companioni

**Affiliations:** 1 IdIHCS, CONICET-National University of La Plata, La Plata, Argentina; 2 Center for Studies of the Cuban Economy, University of Havana, Havana, Cuba; 3 Department of Sociology, University of Havana, Havana, Cuba

**Keywords:** Argentina, care, career trajectories, Cuba, gender, social inequalities, wellbeing

## Abstract

This article presents a comparative analysis of the employment and caregiving trajectories of women from different social classes in Cuba and Argentina, framed within the broader context of persistent gender and class inequalities in Latin America. Based on 24 biographical interviews, the study shows that women’s increasing participation in paid employment coexists with a disproportionate burden of caregiving responsibilities, generating tensions and constraints that affect their access to and retention in the labor market. Although both countries maintain state protectionist welfare systems, their structural differences—a socialist model with strong state involvement in Cuba and a capitalist model characterized by labor market segmentation in Argentina—do not prevent families, and particularly women, from continuing to serve as the primary providers of wellbeing. The findings reveal that women across social classes in both contexts adopt similar strategies to navigate the double burden of paid work and caregiving, illustrating the resilience and adaptability required to manage these overlapping demands. The analysis highlights the interplay between macrosocial structures such as welfare systems, mesosocial dynamics including labor markets and care policies, and microsocial dimensions shaped by individual trajectories. These multilevel interactions demonstrate how inequalities emerge at the intersection of gender, class, and care work. The article concludes by underscoring the need for comprehensive and co responsible public policies that recognize care work as a right and promote shared responsibility among the state, the market, families, and communities. Strengthening social protection systems in this direction is essential to addressing entrenched inequalities and advancing toward more equitable and sustainable models of care.

## 1 Introduction

Latin America continues to exhibit high levels of inequality, though the intensity and dynamics of this phenomenon vary across countries, with some progress observed in the past decade (Amarante et al., 2023). This heterogeneity is evident in the two cases analyzed in this article: Argentina and Cuba. In Argentina, inequality has followed a fluctuating trajectory—declining in the early years of the 21st century, then worsening from 2015 onward, a trend exacerbated by the COVID-19 crisis and subsequent economic policies (Maurizio et al., 2022). The period beginning in 2003, known as the “post-convertibility” era, encompasses two distinct stages: one marked by incipient industrialization and redistributive policies under the Kirchner administrations (2003–2015), and another characterized by the return of neoliberal reforms under the Macri government (2016–2019), a cycle that persists today (Muñiz Terra and Ambort, 2026).

In Cuba, although official income inequality data are not publicly available, various studies indicate a steady increase since 2010 (Zabala and Echevarría, 2020). The situation deteriorated further in the aftermath of the COVID-19 pandemic and the monetary reform of 2021, both of which intensified the economic crisis and inflation. U. S. sanctions, reinforced under the current administration, have compounded these difficulties, worsening living conditions for the Cuban population. While the state continues to uphold social protection programs, recent research highlights the diversification and growing complexity of inequality, disproportionately affecting older adults, women, youth, and households without access to remittances or employment in dynamic sectors (Zabala and Echevarría, 2025).

Inequality in the region is a structural and multidimensional phenomenon, manifesting in social, economic, and gendered forms. Women have been particularly affected, as their increased participation in the labor market intersects with the persistence of family caregiving responsibilities. These processes have been interpreted as part of a “silent revolution” (Goldin, 2006), reflecting the profound transformations in the relationship between work and family. According to CEPAL (2010), seven out of 10 women of reproductive age participate in the labor force, and an increasing number live in female-headed households—many of them single-parent—where caregiving responsibilities continue to fall disproportionately on women.

This article addresses two interrelated analytical problems. First, the sustained growth of women’s labor market participation generates tensions within traditional, still highly feminized, care arrangements. Women’s massive incorporation into paid employment occurs in contexts where caregiving responsibilities remain almost exclusively theirs, forcing them to reconcile work and family demands with highly unequal resources and opportunities. These tensions are strongly stratified: the ability to balance work and family, access institutional support, and delegate reproductive labor varies significantly by social position (CEPAL, 2010). Second, caregiving needs act as a structural constraint on women’s access to, retention in, and continuity of employment, reproducing inequalities across the region. The burden of care not only strains career trajectories but can directly restrict them, particularly among women with limited economic, educational, and social capital.

Taken together, these dynamics reveal that gender and care inequalities are inseparable from broader labor and class inequalities and from the structural transformations underway in the region. Addressing them requires a comprehensive, multidimensional perspective. Within this framework, the article analyzes the configuration of social inequalities from a comparative qualitative perspective in Cuba and Argentina—two countries with distinct societal systems, one socialist and the other capitalist. Specifically, we ask whether, despite structural differences, similarities in social organization can be identified, how they are expressed, and why. To explore these questions, we draw on 24 biographical interviews (12 per country) with women from service, middle, and working-class backgrounds. These interviews trace labor and caregiving trajectories across formal and informal/precarious employment, adopting a comparative biographical approach. This perspective highlights the intertwining of macrosocial processes (welfare regimes), mesosocial processes (labor market policies and care arrangements), and microsocial processes (individual trajectories). Ultimately, the study seeks to interpret the subjective mechanisms through which inequalities are experienced and reproduced in each national context and in comparison.

## 2 Research on social inequalities, women’s work trajectories and the social organization of care in Cuba and Argentina: some background

In Latin America, the study of social and class inequalities has primarily advanced through quantitative research examining intergenerational and intragenerational social mobility, as well as the effects of economic development, equality of opportunity, and social justice on class structures (Benza, 2016; Chávez Molina, 2013; Dalle, 2010; Espinoza et al., 2021; Kessler and Espinoza, 2003; Pla, 2016; Solís and Boado, 2016). Complementary qualitative studies have explored individuals’ representations, decisions, and career paths, focusing on how they experience and interpret their position within the social structure (Mac-Clure et al., 2014; Méndez and Gayo, 2007).

Research on care work and the persistence of gender inequalities—particularly regarding the distribution of unpaid care and its recognition—has largely concentrated on documentary analyses of worker protection policies and statistical data on the social organization of care. Some studies have adopted a comparative perspective across Latin American countries (Bundlender, 2010; Amarante and Rossel, 2017; Campaña et al., 2017; Domínguez Amorós et al., 2019, 2021; CEPAL, 2021; Muñiz Terra et al., 2026), while others have examined national cases through diverse theoretical lenses, including the economics of care, welfare and care systems, care as a right, and the ethics of care (Batthyány et al., 2024; Salvador, 2011; Munevar and Pineda, 2020; Araujo and Hirata, 2020; Arriagada, 2020).

In Cuba, research on women’s career trajectories highlights persistent social and occupational inequalities despite high educational attainment and historical incorporation into state employment. These inequalities are linked to the sexual division of labor, sectoral segmentation, and economic transformations since the 1990s. Studies reveal trajectories marked by women’s concentration in lower-status occupations, the disproportionate burden of domestic and care work, racial inequalities limiting access to better-paying jobs, and the significant role of the informal economy (Romero, 2012; Abreu Sacre, 2016). More recent literature emphasizes women’s growing agency in negotiating career paths amid economic crisis, the expansion of self-employment, and transnational mobility, identifying both upward trajectories associated with educational capital and migration networks, and unstable or downward trajectories linked to the deterioration of state employment and the feminization of informal activities (Espina, 2008; Andaya, 2014).

Studies on care in Cuba show that, since the second decade of the 21st century, the issue has gained visibility in research and specialized publications, consolidating as a politically relevant public concern (Romero, 2020). Key themes include co-responsibility as a factor in women’s empowerment (Romero, 2014; Alvarez, 2015), the social organization of care in relation to resource and time poverty (Lara, 2011), the family as an adjustment mechanism between demand and supply of state services (Álvarez, 2010; Alvarez, 2014; Gross, 2015; Domínguez, 2019), state institutions dedicated to care (Roque et al., 2015; Fusté et al., 2018), and the role of new economic actors, including the informal economy (Romero, 2014; Abreu Sacre, 2016; Hidalgo et al., 2016). Research between 2000 and 2020 (Romero, 2020) also recognizes an emerging interest in conceptualizing care as a system, promoting holistic approaches (Proenza, 2013; Laire, 2016; Azcuy et al., 2018; Gordillo, 2020). These works underscore the need for stronger articulation among the state, family, market, civil society, and community, as well as the mainstreaming of gender equality and rights perspectives in both research and policy evaluation (Álvarez, 2005; Montano and Calderón, 2010; Proveyer et al., 2011; Fleitas and Romero, 2012; Más, 2018; Gross and Peña, 2018; Romero, 2019; Lazcano and Colina, 2020).

In Argentina, qualitative studies of occupational inequalities have examined how social classes experience and interpret their position within the social structure (Gutiérrez and Mansilla, 2015) and the subjective mechanisms of social mobility (Muñiz Terra et al., 2021; Muñiz Terra and Ambort, 2024). Research on women’s career paths has addressed specific groups, including migrant and rural women (Ambort, 2024), women in the fishing industry (Cutuli, 2009), and textile workers (Muñiz Terra et al., 2014). By contrast, studies analyzing the relational configuration of class structures—understood as spaces where class and gender inequalities intersect—remain limited (Connell, 2005; Krause, 2016; Riveiro, 2017).

Most research on care in Argentina has focused on the distribution of responsibilities among the state, market, families, and community. These studies highlight the absence of a comprehensive approach to care policies and the fragmented provision of services, whether linked to labor rights of formal workers (Pautassi et al., 2004) or to state programs targeting the unemployed and those in informal or precarious employment (Esquivel, 2012). Policies include child allowances, early childhood care initiatives (Faur, 2014), and benefits for older adults or people with disabilities (Borgeaud Garciandía, 2020). Scholars agree that limited public coverage forces households to resolve caregiving needs independently, often relying on the service market or, more commonly, unpaid care provided by women within families (Batthyány et al., 2024), thereby reproducing socioeconomic and gender inequalities (Rodríguez Enríquez and Marzonetto, 2015).

Recent research has incorporated gender perspectives enriched by intersectional approaches, considering the interplay of gender, class, and race inequalities in the workplace (Kergoat, 2010). Nevertheless, despite these advances, a significant gap remains in qualitative, comparative regional studies that integrate analyses of work trajectories, care work, and class structures in Latin America.

## 3 Theoretical and methodological perspectives

In this article, career paths will be conceived as those transitions configured over time that result from the interplay between the external world’s opportunity structure (macrosocial scales: welfare regimes and meso-institutional scales: care policies and labor markets) and the actions and decisions of social actors (microsocial scale: configuration of subjective trajectories) (Bertaux, 2005; Pujadas Muñoz, 1992; Muñiz Terra, 2012). Each of these scales is composed of distinct dimensions (in our case: work and care) that are often interconnected. The way in which the multiple scales and their dimensions are linked gives rise to social inequalities that materialize in career paths located in different positions within the social structure (Muñiz Terra, 2021). On the other hand, when we talk about care, we are referring to the social organization of care as “a set of practices, institutions and social relations through which a society distributes, manages and carries out care tasks, involving actors such as the State, the market, families and community organizations. This concept encompasses both paid and unpaid care, and focuses on social co-responsibility, gender equity and the sustainability of wellbeing” (Batthyány et al., 2024). Thus, in this article we analyze how the dimensions of social welfare (labor and care regimes and policies) are articulated with the development of women’s work and care trajectories in different positions within the social structure, thereby shaping social inequalities. These positions are identified according to the notion of social classes proposed by Erikson et al. (1979), who argue that social classes are relational, meaning they are constructed through multiple interactions in the labor market, within which different employment relationships are established. Specifically, this stratification aims to account for two distinctions: between those who own the means of production and those who do not, and, regarding the latter, the type of relationship with their employer. Thus, the central difference lies between positions regulated by an employment contract and those regulated by a “service” relationship. Based on these criteria, they propose the existence of three social classes: service, middle, and working, with different strata (see Table 1). In our study, we revive this tradition and construct a stratification based on the relationships between the three social classes and the mobility that women develop, primarily between the different positions within the macro-classes.

**Table 1:** Class scheme.

I. Professionals, administrators and senior managers	Class of services
II. Professionals, administrators and lower-level managers, technicians, managers of small industrial establishments	
IIIa. Non-manual routine upper-level employees (administration)	Intermediate class
IIIb. Lower-level routine non-manual employees (sales and service)	
VAT. Small business owners without employees	
IVb. Small landowners without employees	
IVc. Small proprietors and other self-employed workers in primary production	
V. Lower-level technicians and supervisors of manual workers	Working class
VIa. Unskilled manual workers (non-agricultural)	
VIIb. Unskilled manual laborers (agricultural)	

Source: Erikson et al. (1979).

In terms of methodology, this article falls within the framework of the Comparative Biographical Perspective (Muñiz Terra, 2018a, 2026). Specifically, it presents two biographical case studies of the work and caregiving trajectories of women from different social classes in Cuba and Argentina who experience transitions between formal and informal/precarious employment in each country. These studies are part of a broader research project that aims to understand the configuration of social inequalities related to class and caregiving in 10 Latin American countries from a comparative biographical qualitative perspective, in order to develop interpretations of the subjective mechanisms that explain these inequalities. The biographical case studies of Cuba and Argentina involved, on the one hand, the application of document analysis to recover the main characteristics of the macrosocial dimensions (welfare regimes) and mesosocial dimensions (particularities of labor markets and care policies), and on the other hand, exploratory fieldwork consisting of 12 biographical interviews per country, conducted in the two capital cities: Havana and Buenos Aires. More specifically, four women were identified from each social class in each country to recover the microsocial dimension (subjective configuration of work and care trajectories). The empirical work involved defining a qualitative sample in each case, configured according to the following criteria: that the interviewees were women, between 35 and 45 years old, with at least 30 h of employment per week, in formal, informal, or precarious jobs, and that they were self-employed or employed. Furthermore, and to adjust for subsequent comparability, it was defined that the interviewees should belong to the following sectors of activity (Table 2).

**Table 2:** Sectors of activity according to social class.

Class of services	- 2 university professors
	- 2 managers of medium-sized companies in the private service sector
Intermediate class	- 2 female administrative workers in the public sector without employees under their supervision
	- 2 small business owners with 1 employee in charge
Working class	- 2 domestic workers
	- 2 caregivers for children or the elderly

Source: Author’s own work.

The interviews used the same interview guide (Appendix 1), which was developed collaboratively for all case studies in the larger research project. Additionally, to facilitate biographical comparisons, 12 biographical grids related to work (Muñiz Terra, 2021) and caregiving were applied in each country. Specifically, semi-structured biographical interviews were conducted, consisting of two interconnected phases: an initial phase in which a narrative of the participants’ work histories was constructed, and a subsequent phase in which a dialogue was established regarding their caregiving trajectories. At each phase, the biographical grid, which captures the interviewees’ work transitions (Appendix 2) and caregiving transitions (Appendix 3), was initially applied. This was followed by a discussion of the developed script, gathering information on various dimensions of their trajectories, such as: the characteristics of the jobs held, the social capital used and its interfamily transmission, the evaluations of the occupations performed, job separations experienced, caregiving tasks, the domestic distribution of time within households, the birth and raising of children, care for elderly adults, the decisions the women made at different points in their trajectories, and so on. The information constructed was initially systematized through the atlas ti software and then interpreted from the use of biographical grids and an analysis of biographical events and bifurcating moments (Muñiz Terra, 2018b). In particular, given that the research was framed within a comparative biographical perspective, four distinct types of comparisons were made: intra-scale, intra-configurative (within each case), and inter-scale and inter-configurative (between cases) (Muñiz Terra, 2018a, 2026). The intra-scale comparison involved an internal comparison within each of the biographical case studies. The aim was to examine the trajectories of each woman from the different social classes in the case studies by country and make a synchronic and diachronic comparison of their particularities, considering the importance that the macro, meso, and micro-social scales acquired in each of them. The intra-configurative comparison involved contrasting the particularities assumed by the articulation/tension of the macro, meso, and micro dimensions in each of the interviews within each class and between classes. Both comparisons will be presented in sections 3.1, 3.2, and 3.3. Interscalar comparison involved comparing the macrosocial, mesosocial, and microsocial scales of the cases with one another. Finally, interconfigurational comparison required comparing, among the cases, the diachronic configuration that arises through the articulation/tension present in the macro, meso, and microsocial scales in each biographical case study. These comparisons will be presented in section 4, Discussion, of this article.

## 4 Results

### 4.1 Family-oriented welfare regimes in Latin America: Cuba and Argentina as case studies

As numerous studies have shown, Latin America continues to exhibit high levels of inequality (CEPAL, 2010; Lustig, 2017; CEPAL, 2024). These inequalities manifest in diverse economic and social contexts and reflect significant structural heterogeneity (Salvia, 2013). They are evident in labor markets characterized by high unemployment, informality, and job insecurity, as well as in weak, inefficient, or absent social policies. In this scenario, families have become the primary providers of social support and protection.

This dynamic has led scholars to classify Latin American welfare regimes as familial (Martínez Franzoni and Sánchez-Ancochea, 2018), given the widespread “familialization” of welfare. In most countries, the weaknesses of labor markets and public policies mean that families—and within them, women—absorb the bulk of social risks and caregiving responsibilities. From a sociological perspective, wellbeing in Latin America is understood as a response to risks and uncertainties articulated across three interconnected spheres: (1) markets (labor and service supply, including care services), (2) the state (labor protection, social security, compensatory policies), and (3) families (support networks, domestic and care work). Within this framework, welfare regimes in the region can be differentiated into:

- State-productivist regimes (Costa Rica, Chile, Uruguay), marked by strong state involvement oriented toward productive development.
- State-protectionist regimes (Argentina, Brazil, Cuba, Mexico), characterized by significant state intervention focused on social protection.
- Family-oriented regimes (much of Central America and the Caribbean, e.g., Nicaragua, Honduras, Guatemala, Dominican Republic), where families predominate due to weak state and market roles (Martinez-Franzoni, 2008).

Focusing on Cuba and Argentina, both countries generally exhibit protectionist state regimes. They provide a degree of protection through state spending and broad social policies, but with segmentation and unequal reach: formal workers are typically covered by social security, while informal workers remain excluded. Families thus assume responsibility for those left outside the protection system. Despite this shared classification, important differences emerge, as Cuba’s welfare regime is embedded in a socialist model with strong state centrality, while Argentina’s is shaped by a capitalist model with relatively weaker state presence.

In Cuba, the welfare system has been sustained for over 60 years by state-led social policies, with limited market participation and strong family support. This model has emphasized universality, free provision of services, equality of rights, and guaranteed access to basic benefits in health, education, employment, social assistance, food, housing, and habitat (Álvarez and Máttar, 2004). Until the late 1980s, the state ensured broad coverage and high-quality services, with relatively low family income demands and tax burdens (Brundenius, 2009; Espina, 2001). Preferential treatment was extended to women, children, the elderly, and rural populations (Álvarez and Máttar, 2004; Peña, 2013).

The crisis of the 1990s, however, marked a turning point. The contraction of state employment, reduction of services in non-priority areas, and opening to private management expanded the role of the market and reinforced families—especially poorer households—as key providers of wellbeing (Zabala, 2010). Social programs targeting vulnerable groups, particularly women and youth, multiplied (Echevarría, 2014). In the second decade of the 21st century, reforms under the “updating of the economic and social model” introduced significant changes: the reduction of egalitarian practices, emphasis on economic sustainability, targeting of subsidies, and greater participation of families and communities. Between 2006 and 2018, the decline in social assistance budgets and beneficiaries reflected the erosion of the state’s protective role (Zabala and Echevarría, 2020). The expansion of the private sector, monetary reforms, and the COVID-19 crisis further intensified commodification processes, though the state retained regulatory authority. Since 2019, more than 15 targeted policies have been implemented, yet declining coverage and quality of universal programs reveal a growing reliance on families and markets, with women disproportionately bearing caregiving responsibilities.

In Argentina, wellbeing has historically been shaped by state protection—through interventions and omissions in labor, education, and health—and by family support, with the market playing a secondary role. Labor protections have primarily benefited formal workers, while informal, precarious, and self-employed workers—representing over 40% of the economically active population—remain excluded. Since the enactment of the 1974 Labor Contract Law, formal workers have enjoyed labor rights, social security, and family allowances, but informal workers must rely on family care or market services. To address these gaps, the state has progressively implemented targeted employment policies, family allowances, and pension benefits for vulnerable groups.

In the early 2000s, several employment programs were introduced—such as the Unemployed Heads of Household Plan, the Temporary Employment Program, Manos a la Obra (Hands-on Work), and Más y Mejor Trabajo (More and Better Work)—which provided resources to poor families in exchange for training or community service (Pautassi et al., 2004). At the same time, the Universal Child Allowance and the Universal Pregnancy Allowance were established, offering cash transfers to informal workers contingent upon health checkups and school attendance (Hintze and Costa, 2011). In 2005, the Pension Inclusion Plan was launched, enabling access to a minimum pension through subsidized contributions, a measure that primarily benefited women (Pautassi and Zibecchi, 2013).

Educational wellbeing, crucial both for children’s development and parents’ work-life balance, has historically been organized through a universal education system guaranteed by the state. Since the early 20th century, compulsory education was progressively extended to early childhood, primary, and secondary levels, with free provision up to higher education. However, the neoliberal reforms of the 1990s decentralized the system, generating segmentation and inequalities. In this context, targeted and compensatory policies were introduced for vulnerable populations, though they have not succeeded in ensuring quality education across all social sectors (Southwell, 2011).

Healthcare has followed a similarly complex trajectory. After a period of expansion toward universality and free access until the 1950s, the system consolidated into three subsystems—public, private, and social security linked to formal employment. Neoliberal reforms in the 1990s introduced decentralization and targeted services, producing a fragmented and unequal model (Belmartino, 2005). Since then, healthcare policy has attempted to balance universal access with targeted programs for vulnerable groups, adopting a dual strategy that guarantees basic services without altering the segmented structure (Cetrángolo and Devoto, 2002).

Overall, the Argentine welfare system combines a strong state presence with marked segmentation among social groups, expressed in fragmented and overlapping policies (Borgeaud Garciandía, 2020). Until the 1990s, welfare was largely based on social security linked to formal employment and the universal provision of education and healthcare. In recent decades, however, oscillations between protectionist and neoliberal policies have reconfigured welfare through targeted interventions for vulnerable populations. These shifts, combined with labor market transformations and the segmentation of education and healthcare systems, have limited the scope of social protection. As a result, large sectors of the population remain insufficiently covered, and the most vulnerable families—particularly women, who lack universal rights and the resources to outsource care—must manage wellbeing under increasingly disadvantaged conditions.

### 4.2 The labor market and the social organization of care in Cuba and Argentina

Social wellbeing depends largely on labor market participation and effective access to social services; thus, the degree of formality or informality in employment, together with unequal access to education and healthcare, are key to understanding positions within the social structure. Likewise, care work within households functions as a central space for the production and reproduction of inequalities. Analyzing the characteristics of the labor market alongside the social organization of care therefore makes it possible to identify the opportunities and constraints faced by different social classes in each country.

In Cuba, employment policy established in 1959 was structured around guaranteed employment, equality, and non-discrimination, positioning the state as the primary job creator for more than five decades (Instituto de Estudios e Investigaciones sobre Trabajo (IEIT), 2004). Until the 1980s, the narrow wage range (4.5 to 1) gave wages a significant redistributive role (Llópiz Hernández, 2008). However, the population boom of the 1960s generated a labor surplus by the 1980s, which was absorbed through workforce underutilization and the expansion of the service sector (García and Piñeiro, 2011). Women’s labor force participation remained conditioned by the persistent burden of domestic work, prompting the creation of programs and services for dependent groups—children, the elderly, and people with disabilities—that can be seen as early mechanisms of work-life balance.

The crisis of the 1990s marked a turning point: the state reduced its activity, while the non-state sector expanded through joint ventures, cooperatives, and self-employment, though labor protections remained under state control (Echevarría, 2014). This transition shifted the system from one based almost entirely on state salaries to one characterized by diversified incomes and widening wage gaps (Espina, 2001). At the same time, the deterioration of social programs reinforced the domestication of caregiving, as services previously guaranteed by the state—such as boarding schools, day centers, and home care—were weakened or eliminated (Romero Almodóvar, 2010). Efforts in the 2000s sought to restore the state’s central role in social protection and expand care services, but caregiving responsibilities continued to fall disproportionately on women.

From the late 2000s onward, reforms driven by efficiency and productivity concerns, as well as the disproportionate ratio of social spending to GDP growth, reshaped the system. Within the framework of the “updating of the economic and social model,” the state downsized public sector employment—dismissing 1.5 million workers—and promoted diversification of non-state employment. This marked a tacit departure from the principle of full employment and reduced the state’s role as the primary source of income. The contraction of the social welfare budget was one of the clearest indicators of this transformation.

With the expansion of non-state employment, the state ceased to be the nearly exclusive employer and provider of income and protection. Social policy increasingly emphasized market mechanisms and family resources, eliminated generalized subsidies, and prioritized targeted spending (PCC, 2016; PCC, 2017). This produced a more selective welfare model, transferring responsibilities to families and deepening inequalities due to heterogeneous starting conditions. The market emerged as a relevant actor in care provision, though without displacing the family-centered principle that continues to guide social policy in cases of greatest vulnerability.

In the third decade of the 21st century, the economic crisis—exacerbated by COVID-19, the tightening of the U.S. embargo, and the implementation of the Tarea Ordenamiento reform plan—created intense pressures: inflation, shortages of essential goods, and exchange rate instability weakened wages as the primary means of accessing wellbeing. Although wage increases were introduced in strategic sectors in 2024, labor force participation fell to 63% in 2023, with women’s participation at just 51.2%. Nearly 20% of the employed population remained in the informal sector, further increasing job insecurity. In parallel, the 2024 approval of the National System for Comprehensive Care for Life sought to place care at the center of the development model—based on principles of shared responsibility and recognition of care as work—but faces significant financial and implementation challenges at the local level.

In summary, while the Cuban labor market continues to exhibit strong state regulation, its role has diminished in the face of private sector dynamism and the growing importance of the market in welfare provision. Care responsibilities, meanwhile, have increasingly shifted toward families, particularly women, marking a transition from a state-run, family-oriented welfare system to one where the market plays a more prominent role. In Argentina, the neoliberal policies implemented in the mid-1970s altered the previous protectionist model and produced profound transformations in the class structure (Jiménez Zunino, 2011). In the following decades, the heterogeneity of the labor market deepened, characterized by a small core of formal and stable jobs and a wide range of precarious and informal occupations (Salvia, 2013). The structural reforms generated contradictory patterns of mobility: upward mobility toward professional and skilled worker occupations coexisted with the relegation of administrative and unskilled jobs to the informal sector, along with a general decline in real wages (Kessler and Espinoza, 2003).

In 2001, Argentina reached its most critical indicators: 18% unemployment, 40% informality, and over 50% poverty (Trujillo, 2017). The crisis triggered a social explosion that ended the neoliberal cycle and ushered in a new political and economic model in 2003. During the Kirchner administrations (2003–2015), redistributive policies and an incipient process of reindustrialization improved labor indicators, though without altering the heterogeneous productive structure. Since 2016, however, a new cycle of adjustment policies favored concentrated economic sectors (Wainer, 2018; Trujillo, 2017), further deteriorating the occupational structure. Following the pandemic, a brief recovery gave way—with the change of government in 2023—to renewed stagnation in formal employment, rising informality, multiple jobholding, declining incomes (Rodríguez de la Fuente, and Sosa, 2025), the emergence of poor registered wage earners (Poy, 2024), and a sustained increase in inequality (Vera et al., 2025).

In response to growing informality and poverty, the state expanded its intervention through social policies in the 2000s. Conditional transfer programs such as the Universal Child Allowance (AUH) and the Pregnancy Allowance targeted unregistered workers and vulnerable families (Pautassi et al., 2004). The Pension Moratorium allowed nearly 4 million people—mostly women—to access minimum pensions without sufficient contributions (Pautassi and Zibecchi, 2013).

Regarding the social organization of care, Argentina has historically lacked an active and comprehensive policy. Access depends on labor rights restricted to formal workers, limited market strategies, and family or community arrangements. Since the enactment of the 1974 Labor Contract Law, care has been partially guaranteed through maternity protection (90 days of leave), paternity leave (2 days), breastfeeding breaks, specific licenses, unregulated childcare centers in large companies, and family allowances. However, this legislation excludes informal workers, self-employed individuals, and freelancers—over 40% of the employed population (Delfino and Fabbioneri, 2021)—perpetuating inequalities in access to care.

To address this unequal distribution, national policies were introduced in the first decade of the 21st century. In 2009, the AUH was created, followed in 2011 by the Pregnancy Allowance for Social Protection. These programs cover unregistered workers, the unemployed, monotributistas sociales (self-employed under the simplified tax regime), and domestic workers earning below the minimum wage. This established a dual system of family allowances: one for formally employed families and another for “vulnerable” households (Pautassi et al., 2004).

Both labor legislation and programs tended to reinforce gender stereotypes, granting rights primarily to women-mothers, who access licenses or allowances and must ensure compliance with conditions such as children’s school attendance and vaccinations (Batthyány et al., 2024).

In childcare, multiple institutional formats coexist: state and private early childhood education, socio-educational programs outside the formal system (e.g., Child Development Centers for vulnerable children), and community-based care spaces (Esquivel, 2012; Pautassi and Zibecchi, 2013). Care for the elderly and people with disabilities is provided through public programs offered by PAMI, the national government, and provincial governments, though coverage remains limited. Home care subsidies are insufficient, and the National Disability Program fails to meet the needs of affected individuals and their families (Borgeaud Garciandía, 2020).

Given these limitations, families—and within them, women—play a crucial role in addressing care needs through unpaid and largely invisible work. With restricted state coverage and limited access to market-based services (daycare centers, day hospitals, etc.), significant differences emerge in how families manage care demands depending on social class. These disparities directly affect women’s opportunities to enter and remain in the labor market.

While statistical tools such as time-use surveys have made this situation more visible (Esquivel, 2012; Domínguez Amorós, 2021), the subjective mechanisms women deploy to reconcile care work with labor market participation remain underexplored. Comparative qualitative studies between countries are particularly scarce, underscoring the relevance of the research presented here.

### 4.3 Intra-scale and intra-configurative biographical comparison: analysis of women’s work and care trajectories in terms of class and gender

The trajectories of the Cuban women interviewed reveal commonalities shaped by the opportunities afforded by universal education policies, which guarantee compulsory schooling up to the secondary level and provide pathways toward technical or higher education. The following analysis highlights, by social class, the particularities of the work transitions and caregiving responsibilities that have defined their career paths.

Working-class women engaged in formal and informal domestic and care work entered the labor market early, typically at age 18, after completing secondary or pre-university education but without pursuing further studies. Their first jobs—secretarial and cleaning positions—were low-skilled, low-paying, and obtained through personal connections rather than prior experience. These roles, though modest, allowed them to contribute to household income, often in contexts of family dysfunction. The decision to forgo further education was driven by the immediate need to improve family living conditions.

Transitions into their current occupations as domestic and care workers reveal strategies of combining multiple jobs before eventually dedicating themselves exclusively to caregiving. While informal employment lacks labor rights, it often provides higher short-term income and greater flexibility than formal jobs. Periods of unemployment were filled with unpaid caregiving and small errands for other households, such as assisting children with homework or school transport. Caregiving responsibilities—first for mothers, siblings, and nephews, and later for their own children—were central to their lives. Early motherhood, around age 20, reinforced this trajectory. Formal employment initially provided maternity leave and childcare services, but the inflexibility of state jobs ultimately led them to informal work. As one interviewee explained: “As a mother, you have to prioritize raising your children above being a woman, a wife, and everything else. Since I was at home, recovering from cancer treatment, I was the one who could best take care of them.” (Interview No. 1).

Middle-class women pursued education up to the pre-university level and entered the workforce at age 18 in state institutions, often through job placement policies or family connections. Echoing Espina (2008) findings, the interviews revealed limited motivation to pursue further opportunities, including self-employment, despite their availability. They did not experience unemployment but supplemented their state jobs with informal domestic work. Job security and flexible hours compensated for low wages, and their income was valued as complementary to that of other household members. Caregiving responsibilities were less pronounced, though some recalled caring for siblings from a young age. For women from single-parent families, maternal support was decisive in enabling their educational and career advancement. As one interviewee reflected: “Since my mother was single and had to work, I had to learn to do everything around the house from a young age (…) my mother was always there for me every day, whenever I had a problem. When I wanted to go to university, I had a lot of support from her because she had two young daughters.” (Interview No. 2).

Service-class women—university professors and a service company manager—completed higher education and entered the workforce in their mid-twenties through state job assignment policies. Their academic trajectories included attendance at elite pre-university institutes (IPVCE), advanced degrees, scholarships from CLACSO, and participation in international exchange programs, particularly in Europe. These experiences enhanced their professional prestige and supplemented family income. One interviewee combined academic work with a managerial role in a private MSME providing care services.

Despite their professional achievements, service-class women reported tensions between demanding workloads and domestic responsibilities. As one explained: “Sometimes many things pile up on me. We also do projects with international organizations that sometimes put a lot of pressure on me because there are the everyday things that every woman caregiver who does domestic work (…) the things I have to do at home, the work for the classes, and there are days when we have an order for 500 jars of natural cosmetics. What I do is separate myself from everything and organize priorities. I think that has always been my technique for organizing myself (…) it’s like a mental map of priorities.” (Interview No. 10).

The interviewees from the middle class and the university professors acknowledged the support they received from their families (mothers and fathers), both in terms of their motivation to study and the financial support they received: “It was important for my family that I reach a certain level of education… but they were not the typical parents who were constantly hovering over me to check my grades” (Interviewee No. 3). In contrast, for those from the working class, studying represented an opportunity to escape their family circumstances and to transform their starting point. In these trajectories, caregiving is primarily centered on motherhood, specifically the care of their own children. Only one interviewee, a university professor, shared her experience of providing care to an adult with a prolonged illness, as well as domestic work, while beginning her entry into the labor market. She described her experiences: “For the first time, I felt like an adult, like I was responsible, (…) I also had to do caregiving at home, that is, with my mother (…) I did not have time to cry, because there was a 13-year-old girl to take care of (…) because I had to take on everything for my mother, everything in the house, and it was a complicated time. It coincided with my first job (…) (Interviewee No. 11). The itineraries reveal three key moments shared by middle-class and service-class workers due to their ties to the public sector. The first moment involves access to 12 months of maternity leave and authorizations for absences during prenatal and postpartum medical appointments, as well as during the first 3 years of their children’s lives. They describe the enjoyment of motherhood without the risk of losing their jobs and with the continued possibility of receiving income. A second phase, identified from the institutionalization of childcare and education with children’s entry into state-run early childhood centers or nurseries, for which a nominal fee was paid, including snacks and lunch, during hours that coincided with the daily work schedule. All the interviewees referred to having received support from their families—mothers, fathers, siblings—and from their children’s fathers during their childhood, enabling them to balance caregiving roles with paid work. They stated, “It’s everyone’s responsibility: my brother’s, who does not have children, my dad’s, my mom’s… we organized ourselves to arrive early: my dad would take him to the center and my mom or dad would pick him up. We’re always focused on him; he’s the center of our lives” (Interviewee No. 1). A third moment is the accompaniment of adolescent children in vocational guidance: “the new generation of the family has imposed on those who come, that they be someone in life, that they study so that they have a future” (Interviewee No. 6) and in the creation of responsibilities: “I know I have done wrong, I never let them do anything at home” (Interviewee No. 6).

The employment and caregiving trajectories of the Argentine women interviewed reveal distinct patterns shaped by social class.

Working-class women engaged in formal and informal domestic and care work typically entered the labor market during adolescence, often while still attending secondary school. Their first jobs—informal factory work or domestic service—were aimed at covering personal expenses and contributing to family income. The demanding hours of these jobs frequently delayed or interrupted their education, leading many to drop out of secondary school and pursue vocational training courses to generate additional income. As one participant explained: “My parents could not afford the books for me to study, or take me to private lessons, because I struggled a lot with chemistry, math, all that stuff. So, they just could not afford it, and well, I left school there, finishing second year of high school.” (Interviewee No. 5).

Their career paths were marked by transitions across various short-term, low-paying, physically demanding jobs—hairdressing assistant, flyer distributor, street vendor—characterized by informality and poor working conditions. Periods of unemployment were filled with odd jobs, as inactivity was not an option. Multiple jobholding was common, often supplemented by participation in state-run social employment programs such as Plan Trabajar or the Universal Child Allowance, which provided income through feminized tasks like cleaning, childcare, or cooking in community kitchens. As one woman described: “On weekdays, when people need a haircut, well, I give them an appointment in the afternoon, after my cleaning job, and I tell them: come to my house at such and such time, and they go and get their hair cut, because I also know a few hairdressing skills.” (Interviewee No. 5).

Caregiving responsibilities began in childhood, when these women assumed early roles caring for siblings and grandparents, and continued throughout their lives. Withdrawal from the labor market during pregnancy and early childrearing was common, driven by both the desire to be present and the economic impracticality of outsourcing care. Later, children’s enrollment in public kindergartens or schools enabled mothers to re-enter the workforce, though always in low-income jobs adapted to caregiving demands. Their narratives highlight the moral weight of maternal responsibility: “I had to divide my time, the day only had 24 h, right? Because you have to be there. Everything is important for my children and I do it for them.” (Interviewee No. 4).

Middle-class women entered the labor market as public-sector employees or self-employed workers, with more stable occupational trajectories. While they initially experienced informality, over time they achieved formal employment or stability in service-sector jobs. As one explained: “I’ve been a hairdresser for 20 years… Nico (her husband) and I have been self-employed for many years… I started as an employee without a contract in a hair salon, and then a long time ago I opened my own salon, and I’ve been here ever since.” (Interviewee No. 1).

Their early labor market entry often coincided with secondary schooling, motivated by the need to cover personal expenses. Some received state scholarships, though these were insufficient, leading to educational delays and late completion of secondary education. Attempts at higher education were often abandoned due to family responsibilities. Multiple employment remained common, as formal or independent jobs with social security did not provide sufficient income.

Caregiving trajectories in this group were shaped primarily by childcare, with less responsibility for elderly relatives due to institutional support (pensions, social security). This reduced their caregiving burden compared to working-class women. Their accounts reveal distinct stages: (a) pregnancy and early months, marked by extended maternity leave and exclusive caregiving; (b) early childhood, with shared caregiving supported by daycare centers; (c) childhood, involving diverse care networks of partners, relatives, and nannies; and (d) adolescence, characterized by children’s growing autonomy and parental guidance. As Julieta explained: “When my daughter was born, everything started to change, because my time is different… she takes time, she needs time, and it’s time I want to give her. So I stopped doing a million things and started working less.” (Interviewee No. 1).

Service-class women—university lecturers and small business directors—began their careers during their university studies, often through internships or informal arrangements with low wages. Their subsequent trajectories were professional, supported by higher education and, in some cases, postgraduate qualifications. Early experiences were exploratory and precarious, but eventually led to stability. Family and partner support was crucial, both financially and emotionally. As one explained: “I had already moved in with my husband, and that was incredibly important when I lost my doctoral scholarship, because he supported me financially and also kept me company during that period of uncertainty.” (Interviewee No. 9).

These women’s trajectories reveal greater capacity for choice compared to middle-class workers, both in vocational decisions and in negotiating working conditions. Their accounts highlight how material and emotional support from family networks enabled them to pursue professional careers with relative autonomy.

For service-class women, multiple jobholding was also present in their trajectories, though primarily associated with vocational motivations or the desire to explore new paid work experiences that could open future opportunities. While the search for additional income was part of the rationale, these positions were sporadic and valued as unique opportunities that enriched their lives by providing experience, new relationships, and professional history.

In terms of caregiving, most interviewees did not assume responsibilities until becoming mothers. In their families of origin, childcare and eldercare were managed by other women in the household (primarily mothers), supplemented by daycare centers, senior living facilities, or the hiring of nannies and caregivers. Motherhood generally occurred in adulthood, around the age of 30, after completing university studies and establishing professional careers that provided satisfaction and economic stability. At this stage, they faced persistent tensions between the time dedicated to work and the care of their children, a negotiation that evolved over time.

Their caregiving trajectories reveal distinct phases supported by institutional and family arrangements that enabled them to combine professional careers with motherhood. During pregnancy and the first months of their children’s lives, they accessed maternity leave through formal employment or company arrangements, with caregiving responsibilities shared between themselves and their partners.

In the second stage, corresponding to early childhood, they relied on what one interviewee described as a “caregiving family” (Interviewee No. 9), composed of partners, grandmothers, private daycare centers, and, most importantly, nannies. These caregivers—formally or informally employed and present daily for several hours—played a central role in childcare and domestic tasks, generating strong emotional bonds and becoming integral to family life.

The third stage, during school age (4–12 years), involved new forms of care organization depending on the type of schooling chosen. In single-shift schools, the care network remained stable, while double-shift schools reduced the need for nannies or shifted their tasks toward household maintenance. During this period, some women also began to manage care for elderly parents, though this responsibility focused more on coordinating institutional services than on providing direct care.

Finally, in adolescence and early adulthood, the “caregiving family” gradually disintegrated as children achieved greater autonomy. Caregiving at this stage centered on emotional support, guidance, and accompaniment in decision-making, rather than daily tasks.

Taken together, these narratives highlight the morality that guides service-class women’s understanding of “adequate” care and the weight of expectations surrounding their role in family life. Their accounts reveal a persistent tension and ongoing search for balance between professional aspirations, maternal responsibilities, and moral obligations in the daily organization of household care.

## 5 Discussion. Interscalar and interconfigurative comparison: the diachronic sedimentation of welfare regimes, labor markets, the social organization of care and trajectories

The diachronic configuration of social inequalities in work and care within Cuba and Argentina provides interpretative axes that allow us to contrast both cases in a comparative key. By examining interscalar dynamics (macro, meso, and microsocial levels) and interconfigurational articulations (the interplay among these levels), several similarities and differences emerge.

First, at the macro level, Cuba’s family-based welfare system—historically sustained by a strong socialist state presence—has gradually moderated its social assistance role. This trend is evident in the employment and caregiving trajectories of younger women, who face fewer guarantees than previous generations. In contrast, Argentina’s family-based welfare system, embedded in a capitalist model, exhibits a relative state presence and increasing segmentation, fragmentation, and targeting of social assistance, a recurring theme in the interviews.

Second, at the meso-institutional level, Cuba shows a gradual decline in employment opportunities and state-provided care benefits for women. The persistent foreign economic blockade, despite countercyclical strategies, has eroded the full employment and care policies established after the revolution. As a result, the children of the women interviewed face fewer opportunities for stable, formal employment and reduced access to maternity care compared to their mothers. In Argentina, the labor market has shifted from a mid-20th-century structure with high levels of formal employment to one characterized by precariousness, informality, and poverty. Care policies remain limited, largely tied to maternity leave and pregnancy care through formal employment. Consequently, women in informal jobs or poverty lack access to these benefits and must rely on a deteriorating public health system. Beyond this stage, childcare and adolescent care fall primarily to families, as reflected in the arrangements described by interviewees in both countries.

Third, at the microsocial level, women’s representations, strategies, and decisions reveal both convergences and divergences across social classes. In terms of education, Cuba’s universal compulsory secondary education policy ensures completion for the entire population, enabling women to enter the labor market after age 18. In Argentina, although compulsory education is also universal and graduation rates have improved, completion is not always achieved due to economic and educational difficulties. As a result, Argentine women often enter the labor market during adolescence, while still attending secondary school, and many drop out to dedicate themselves almost exclusively to work.

Working conditions also differ. In Cuba, working-class women’s first jobs are typically formal positions in the public sector, whereas in Argentina they are informal, private-sector roles. In both contexts, these jobs are accessed through family or social networks and provide low wages. Employment trajectories diverge over time: in Cuba, additional jobs in domestic or caregiving roles eventually become the primary source of income, as women leave formal employment with labor rights but low wages for better-paying informal care work. In Argentina, transitions remain largely informal and low-income, forcing women to rely on multiple jobs to sustain their families.

In summary, while both Cuba and Argentina share the predominance of family-centered care networks and the persistence of gendered inequalities, their trajectories diverge in terms of institutional guarantees, labor market structures, and the timing of women’s entry into the workforce. Cuba’s socialist welfare regime historically ensured universal access but has weakened under economic pressures, while Argentina’s capitalist regime has long been segmented, leaving large sectors of women dependent on family and informal arrangements.

The diachronic analysis of social inequalities in work and care across Cuba and Argentina highlights both convergences and divergences when examined through interscalar (macro, meso, and microsocial) and interconfigurational (articulation across levels) perspectives.

Working-class women in both countries begin their caregiving trajectories early, often during childhood and adolescence. Yet the conditions of motherhood diverge sharply. Cuban women typically become mothers while employed in the formal sector, with access to maternity leave and state-run early childhood care. Argentine women, by contrast, have historically worked in the informal sector, lacking maternity leave and institutional childcare. In both contexts, however, motherhood is emphasized as a central responsibility, shaping career choices and reinforcing the expectation that women “must” prioritize care for their own children.

Middle-class women show overlapping educational attainment, generally completing 12 years of schooling. In Argentina, some attempt higher education at public, tuition-free universities, but often abandon studies due to academic difficulties and financial constraints. This contrasts with Cuba, where state educational policies actively support completion. Employment trajectories also differ: in Cuba, job placement occurs after secondary school through state institutions, often mediated by family or social networks; in Argentina, women enter the labor market during secondary school, initially in informal jobs, before transitioning to formal or self-employed work. Caregiving responsibilities in both countries focus primarily on children rather than elderly relatives, thanks to institutional support (pensions in Argentina, public programs in Cuba). While maternity leave is available in both contexts, Cuban women benefit from state childcare services, whereas Argentine women rely on family networks and sporadic hired help. Across both cases, women express dissatisfaction with wages but value the stability that allows them to organize childcare, which they perceive as their personal responsibility.

Service-class women in both countries complete university studies without entering the workforce during their education. Cuban women benefit from state guarantees of higher education and job placement, while Argentine women rely on free public universities but lack guaranteed employment, often beginning with informal or precarious jobs before stabilizing professionally. In both contexts, multiple jobholding is common, not only to increase income but also to enhance prestige and professional recognition. Caregiving trajectories are centered on childcare, supported by maternity leave and shared responsibilities with partners and relatives. The notion of the “caregiving family” emerges in both cases, though in Argentina it explicitly includes the hiring of nannies—formally or informally—who become integral to the caregiving network, a practice not mentioned by Cuban women.

In summary, while both Cuba and Argentina share the predominance of family-centered care networks and the moral weight of motherhood in shaping women’s trajectories, their institutional contexts diverge. Cuba’s socialist welfare regime historically guaranteed universal access to education, employment, and childcare, though these guarantees have weakened over time. Argentina’s capitalist regime, by contrast, has long been segmented, with limited institutional support, leaving women more dependent on informal work and family arrangements (Tables 3, 4).

**Table 3:** Comparison of interscalar and interconfigurational biographical differences in work and care between Cuba and Argentina.

Dynamic differences at the macrosocial level Welfare Regimes	CUBA	ARGENTINA
All social classes	Family-oriented with a strong state presence in health, education, and assistance	Familialist with extractive capitalist state presence, segmented and focused
Dynamic differences at the level Mesosocial: labor market and social organization of care	CUBA	ARGENTINA
All social classes: Labor Market	Full employment guaranteed by the State but with a tendency toward a shrinking of the public role in job creation and the granting of low wages	Labor market with a shift from a high percentage of formal employment to increasing job insecurity and a gradual increase in informality and structural poverty
All social classes: Institutional social organization of care	National state programs for dependent care groups, the elderly, people with disabilities and early childhood, with a tendency toward deterioration of state benefits but with the design of an incipient national care system	The absence of a national care system, which is generally guaranteed by women in families, with benefits for formal workers such as maternity leave, public pregnancy care for informal workers, and various early childhood care institutions.
Dynamic differences at the microsocial level Work and care trajectories	CUBA	ARGENTINA
Working class		
High school graduation	YES	NOT ALWAYS
Educational system that guarantees secondary school completion	YES	NO
First job placements	Formal	Informal
Age of first entry into the workforce	18 or older	Around 16 or more
Goal of first job placement	Family collaboration	Personal expenses and family contributions
Medium-term work	Informal (leaving formal work due to low income)	Informal (impossibility of formality)
Access to maternity leave	YES	NO
Access to state-run early childhood care	YES	NO
Intermediate class		
Starting and dropping out of higher education	NO	YES
First job placements	Formal	Informal
Educational system that guarantees secondary school completion	YES	NO
Moonlighting	NO	YES
Access to state-run early childhood care	YES	NO
Class of services		
Entering the labor market during university	NO	YES
First job placement	Formal (with state support)	Informal
Medium and long-term career path	Formal	Formal
Early childhood care trajectory	With state support	With private support (nannies or daycare centers)
Care pathways for early childhood, childhood and adolescence/youth	With state support	Caregiving family with nannies
Recognized institutional support (state support for higher education)	YEAS	NO

Source: Own elaboration.

**Table 4:** Identification of interscalar and interconfigurational biographical coincidences of work and care between Cuba and Argentina.

Dynamic coincidences at the macrosocial level	CUBA and ARGENTINA
All social classes	Familialist–protectionist welfare regime
Dynamic coincidences at the mesosocial level	CUBA and ARGENTINA
All social classes: Labor market	Trend toward deterioration of the employment situation in the labor market.
All social classes: Institutional social organization of care	Lack of an integrated policy for the social organization of care. However, maternity licenses are available. Public care for early childhood.
Dynamic coincidences at the microsocial level	CUBA and ARGENTINA
Working class	
Income from first job placements	Low
Type of access to first job	Family or friendship networks
Multiple jobs throughout working life (domestic and care work)	To improve income
Care in childhood and adolescence period	Grandparents, parents, siblings, nieces and nephews
Care in youth and adulthood	Their own children and those of their ill parents. Self-care
Intermediate class	
Completion of secondary education	In both countries, women complete 12 full years of schooling.
Age of first job placements	18 or more
Type of access to first job	Family or friendship networks
Medium- and long-term career paths	Formal or independent
Access to maternity leave licenses	YES
Care trajectories in childhood/adolescence primarily under their responsibility	It has not been necessary to take care of grandparents, but on occasions it has been necessary to take care of siblings.
Care pathways in adulthood	Care of their children under their responsibility and the formation of care networks. Care of their older parents
The relationship between Work/care	Balance between work in the labor market and the organization of family care
Service class	
University graduation	YES
Working during university studies	NO
Pursuit of postgraduate studies	YES
Care in childhood/adolescence period	They do NOT take care of grandparents, parents or siblings.
Care trajectories in adulthood	Care network focused on the mother and support from partners and close family members

Source: Own elaboration.

## 6 Conclusion

This article has examined the configuration of social inequalities from a comparative qualitative perspective in two Latin American countries—Cuba and Argentina—whose societal structures differ significantly, being inscribed, respectively, in socialist and capitalist models.

The analysis demonstrates that differences between the societal organizations of both countries prevail over the identified similarities. A certain heterogeneity is evident across the macro, meso, and microsocial dimensions, with each level revealing specific divergent and coincident factors that shed light on the subjective mechanisms shaping the inequalities analyzed.

As summarized in the contrasts at the macro- and meso-social levels are particularly significant. Cuba’s protectionist, family-oriented welfare system reflects a stronger state presence in the establishment of welfare regimes, labor markets, and institutional care, providing educational and employment guarantees—such as access to a first public sector job—that Argentina’s segmented, capitalist welfare system does not offer. In Argentina, welfare provision is more fragmented and targeted, leaving large sectors of the population dependent on family-based arrangements.

At the microsocial level, the subjective strategies employed by women reveal important differences across social classes. In Cuba, working-class women often abandon public sector employment and its associated social security benefits due to low wages, opting instead for informal jobs that provide higher short-term income. In Argentina, working-class women’s trajectories are consistently built in the informal sector, characterized by low-skilled, low-income occupations without access to institutionalized care. Their strategies revolve around “making ends meet,” relying on multiple jobs and maternal caregiving as the primary support system.

Among the middle class, Cuban women pursue stable, formal employment in the public sector, relying on combined family income and focusing primarily on caregiving with strong family support networks. Argentine middle-class women, by contrast, move from informality to formality, supplementing their primary employment with additional jobs to increase income. Without guaranteed institutionalized care, they construct caregiving networks with partners and relatives, occasionally supplemented by hired nannies.

Service-class women in Cuba build their trajectories on public institutional support for education and employment, enabling them to pursue formal careers in the public sector and, in some cases, improve their family’s living conditions. Caregiving is organized through state-provided early childhood services and family networks. In Argentina, service-class women transition from informal to formal employment, gaining recognition and income through professional careers. Their caregiving arrangements are organized around the notion of a “care family,” coordinated by them as the primary responsible actors, with participation from partners, relatives, and hired nannies.

Despite these differences, several broad similarities emerge. Both countries share family-oriented welfare systems, a worsening labor market situation, and care policies largely limited to motherhood and early childhood. Across social classes, women’s trajectories converge in the feminization of care, which assigns them primary responsibility for family wellbeing, and in the morality of care, linked to the normative ideal of “being a good mother.” These cultural mandates condition decisions and generate tensions in balancing work and caregiving.

The findings underscore that the relationship between work and care does not operate as a unidirectional causality—where employment determines care or vice versa—but as a relational and dynamic process. Women across social classes develop situated strategies to reconcile both spheres under structural, symbolic, and moral pressures. The specific forms these strategies take vary according to social position and historical context, shaped by unequal access to resources, institutional policies, and family support networks, as well as by women’s aspirations, experiences, and horizons of mobility.

Taken together, these dynamics reveal that women’s efforts to balance career paths and caregiving responsibilities are always provisional, negotiated within the constraints of structural inequalities and cultural expectations. This constant search for equilibrium constitutes a fundamental analytical key for understanding the configuration of social inequalities in Cuba and Argentina.

## Data Availability

The original contributions presented in the study are included in the article/Supplementary material, further inquiries can be directed to the corresponding authors.

## Author’s note

This article is part of two ongoing projects: the project “Labor Trajectories and Appropriation of Digital Technologies by Different Social Classes in the AMBA (2019-2026): An Analysis of the Pre-COVID-19–Post-COVID-19 Cycle” (Code H992 UNLP) and the project “A New Measure of Socioeconomic Inequalities for International Comparison” (INCASI2) under the Horizon Europe, Marie Skłodowska-Curie Actions (MSCA), Staff Exchange (GA101130456).

## References

[ref1] Abreu Sacre S. (2016). El trabajo doméstico remunerado a domicilio en Alamar: ¿una opción tras la jubilación? Revista Novedades en Población 12, 142–155. Recuperado en 18 de julio de 2026, de http://scielo.sld.cu/scielo.php?script=sci_arttext&pid=S1817-40782016000200013&lng=es&tlng=es

[ref2] Álvarez M. (2005). La familia cubana: políticas públicas y cambios socio – demográficos, económicos y de género. En I. Arriagada y V. Aranda (coords). Cambios de las familias en el marco de las transformaciones globales: necesidad de políticas públicas eficaces. Serie Seminarios y Conferencias No. 42. Santiago de Chile: CEPAL. Disponible en https://repositorio.cepal.org/handle/11362/6785.

[ref9001] Álvarez M. (2010). Género: Teoría y transformación social. Centro de Estudios de la Mujer (CEM): La experiencia cubana. La Habana.

[ref3] Alvarez M. (2014). “Estrategias familiares para el cuidado de los(as) adultos(as) mayores. Estudio exploratorio con 89 familias” in En Mayda Alvarez y Lien Más (comps.), Familia y género. Continuidad y rupturas (La Habana, Cuba: Editorial de la Mujer), 107–112.

[ref4] Alvarez M. (2015). Políticas públicas de cuidado con corresponsabilidad. Novedades en Población, 130–136. Recuperado en 18 de julio 2026 de http://www.scielo.sld.cu/pdf/rnp/v11n21/rnp090115.pdf

[ref5] Álvarez E. Máttar J. (2004). Política social y reformas estructurales: Cuba a principios del siglo XXI. La Habana: CEPAL / INIE / PNUD.

[ref6] Amarante V. Lustig N. Vigorito Z. (2023). The challenge of income inequality in Latin America. CEPAL Rev. No. 141.147-163. https://www.cepal.org/en/publications/69200-challenge-income-inequality-latin-america

[ref7] Amarante V. Rossel C. (2017). Unfolding patterns of unpaid household work in Latin America. Fem. Econ. 24, 1–34. doi: 10.1080/13545701.2017.1282600

[ref8] Ambort M. E. (2024). Una mirada feminista de la “escalera boliviana”. Trayectorias hortícolas de mujeres quinteras en el Gran La Plata, Argentina. Revista Española De. Sociología 33. a241 Recuperado 18 de julio 2026 de: https://dialnet.unirioja.es/servlet/articulo?codigo=9616125

[ref9] Andaya E. (2014). Conceiving Cuba: Reproduction, women, and the state in the post-soviet era. New Brunswick, NJ: Rutgers University Press.

[ref9002] Araujo N. Hirata H. (2020). El cuidado en América Latina. Buenos Aires: Fundación Medifé.

[ref9003] Arriagada I. (2020). Documento caso Colombia (Colección Horizontes del cuidado). Buenos Aires: Fundación Medifé.

[ref9004] Aguilera A. L. Pérez A. C. Doval Y. R. (2018). Propuesta de perfeccionamiento de la política social para el adulto mayor desde la cooperación intersectorial. Revista Novedades en Población 14, 1–8. Recuperado en 18 de julio de 2026, de http://scielo.sld.cu/scielo.php?script=sci_arttext&pid=S1817-40782018000100013&lng=es&tlng=es

[ref10] Azcuy L. Camellón A. Roque Y. (2018). Proposal for improving social policy for older adults through intersectoral cooperation. Noved. Poblac. 14, 1–9.

[ref11] Batthyány Karina Perrotta Valentina Javier A. Pineda Duque (2024) La sociedad del cuidado y políticas de la vida. Ciudad Autónoma de Buenos Aires: CLACSO; México: INMujeres; UNAM; Ginebra: UNRISD, (2024). Available online at: (https://www.clacso.org/wp-content/uploads/2024/04/La-sociedad-del-cuidado.pdf). (Accessed July 16, 2025).

[ref12] Belmartino S. (2005). La atención médica argentina en el siglo XX. Instituciones y procesos. Buenos Aires: Siglo XXI Editores.

[ref13] Benza G. (2016). La estructura de clases argentina durante la década 2003–2013. Kessler En G. (Comp.), La sociedad argentina hoy. Radiografía de una nueva estructura (pp. xx–xx). Buenos Aires: Siglo XXI.

[ref9005] Bertaux D. (2005). Los relatos de vida. Perspectiva etnosociológica. Barcelona: Edicions Bellaterra.

[ref14] Borgeaud Garciandía N. (2020). “Entre desarrollo y fragmentaciones: estudios y panorama del cuidado remunerado en Argentina, en Araujo Guimaraes e Hirata (comp.)” in El cuidado en América Latina: mirando los casos de Argentina, Brasil, Chile, Colombia y Uruguay (Ciudad Autónoma de Buenos Aires: Fundación Medifé).

[ref9006] Brundenius C. (2009). Revolutionary Cuba at 50: growth with equity revisited. Lat. Am. Perspect. 36, 31–48. http://www.jstor.org/stable/27648178

[ref15] Bundlender D. (2010). What do time use studies tell us about unpaid care work? Evidence from seven countries. Nueva York: Routledge.

[ref16] Campaña J. C. Giménez-Nadal J. Molina J. A. (2017). Gender norms and the gendered distribution of total work in Latin American households. Fem. Econ. 24, 35–62. doi: 10.1080/13545701.2017.1282601

[ref9007] CEPAL (2010). La hora de la igualdad: Brechas por cerrar, caminos por abrir. Santiago de Chile: Comisión Económica para América Latina y el Caribe.

[ref9008] CEPAL (2021). Hacia la sociedad del cuidado: Los aportes de la Agenda Regional de Género en el marco del desarrollo sostenible. Santiago de Chile: CEPAL.

[ref9009] CEPAL (2024). Reducir la desigualdad y avanzar hacia el desarrollo social inclusivo en América Latina y el Caribe: desafíos, prioridades y mensajes de cara a la Segunda Cumbre Mundial sobre Desarrollo Social (LC/MDS.6/3). Santiago de Chile: CEPAL.

[ref17] Cetrángolo O. Devoto F. (2002). La salud en las reformas de la política social en Argentina. Santiago de Chile: CEPAL.

[ref18] Chávez Molina E. (2013). Desigualdad y movilidad social en el mundo contemporáneo. Aportes empíricos y conceptuales. Argentina, China, España y Francia. Buenos Aires: Imago Mundi.

[ref19] Connell R. W. (2005). Masculinities. Berkeley: University of California Press.

[ref20] Cutuli R. (2009). Trayectorias laborales precarizadas: mujeres de la industria pesquera marplatense. Revista de la Facultad de Humanidades y Ciencias Sociales 9, 1–20.

[ref21] Dalle P. (2010). Estratificación y movilidad social en Argentina. Revista de Trabajo 8, 59–83.

[ref23] Delfino A. y Fabbioneri, F. (2021). ‘Los cuidados en la agenda democrática: una mirada desde la Ley de Contrato de Trabajo’, Temas y Debates. Revista Universitaria de Ciencias Sociales 41, 135–153.

[ref24] Domínguez Amorós M. [coautores]. (2021). “Social times, reproduction and social inequality at work: contrasts and comparative perspectives between countries” in Comparative analysis of social inequalities between Europe and Latin America. eds. López-Roldán L. Fachelli S. (Springer), 331–360. doi: 10.1007/978-3-030-48442-2_11

[ref25] Domínguez Amorós M. Muñiz Terra L. Rubilar Donoso G. (2019). El trabajo doméstico y de cuidados en las parejas de doble ingreso: Análisis comparativo entre España. Argentina y Chile. Revista Papers 104, 1–38.

[ref26] Domínguez Y. Y R O. (2019). Estudio de seguridad alimentaria familiar en comunidades de Santiago de Cuba: una contribución a la gestión del desarrollo local. En: Colectivo de autores, Ciencia e Innovación Tecnológica. Santiago de Cuba: Editorial Académica Universitaria, pp. 111–118.

[ref27] Echevarría D. (2014). Trabajo remunerado femenino en dos momentos de transformación económica. Revista Temas 80, 45–54.

[ref9010] Erikson R. Goldthorpe J. H. Portocarero L. (1979). Intergenerational class mobility in three Western European societies: England, France and Sweden. Br. J. Sociol. 30, 415–441. doi: 10.2307/589632 20092493

[ref28] Espina M., (2001). The effects of the reform on Cuba’s social structure: an overview. En: Social. Democr., Vol. 15, No., pp. 23–39. https://sdonline.org/issue/29/effects-reform-cuba%E2%80%99s-social-structure-overview (Accessed June 24, 2026).

[ref29] Espina M. (2008). Políticas de atención a la pobreza y la desigualdad. Examinando el rol del Estado en la experiencia cubana. Buenos Aires: CLACSO-CROP.

[ref30] Espinoza V. Rey R. Barozet E. (2021). Incidencia del capital social en el logro ocupacional en Uruguay y Chile. Estudios Sociológicos 39, 395–432. doi: 10.24201/es.2021v39n116.2025

[ref31] Esquivel V. (2012). “El cuidado infantil en las familias. Un análisis en base a la Encuesta de Uso del Tiempo de la Ciudad de Buenos Aires”. En Esquivel V. Jelin E. Faur Y E. (comps.). Las lógicas del cuidado infantil. Entre las familias, el Estado y el mercado (pp. 73–105). Buenos Aires: IDES, UNFPA, UNICEF.

[ref32] Faur E. (2014). El cuidado infantil en el siglo XXI. Mujeres malabaristas en una sociedad desigual. Buenos Aires: Siglo XXI Editores.

[ref33] Fleitas R. Y Romero M. (coords) (2012). Familia, Género y Violencia Doméstica. Diversas experiencias de investigación social. La Habana, Cuba: Instituto Cubano de Investigación Cultural Juan Marinello.

[ref34] Fusté M. Pérez M. Y. Paz L. E. (2018). Caracterización de las redes de apoyo formal al adulto mayor en la Casa de Abuelos del Municipio Camajuaní. Cuba. Novedades en Población 14, 1–12. Recuperado en 18 de junio de 2026, de http://scielo.sld.cu/scielo.php?script=sci_arttext&pid=S1817-40782018000100012&lng=es&tlng=es

[ref9012] García A. Piñeiro B. A. Y. C. (2011). Restructuración del empleo en Cuba: el papel de las empresas no estatales. La Habana: CD Seminario sobre Economía Cubana y Gerencia Empresarial.

[ref35] Goldin C. (2006). The quiet revolution that transformed women’s employment, education, and family. Am. Econ. Rev. 96, 1–21. doi: 10.1257/000282806777212350

[ref36] Gordillo L. (2020). Oportunidades y desafíos para un sistema integral de cuidados. Recuperado de. (https://www.redsemlac-cuba.net/redsemlac/sociedad-y-cultura/oportunidades-y-desafios-para-un-sistema-integral-de-cuidados/ (Accessed September 11, 2025).

[ref37] Gross A. (2015). “El adulto mayor en el escenario migratorio cubano actual. Redes familiares transnacionales vs. vulnerabilidades sociales” in En: Ángela Peña (coord.), Desigualdad y problemas del desarrollo en Cuba (La Habana, Cuba: Editorial Universidad de La Habana), 85–102.

[ref38] Gross A. Y. Peña Á. (2018). La política del cuidado en Cuba. Retos y perspectivas para un diseño multiactoral de cuidado con énfasis en adultos mayores. Universidad de La Habana 286, 155–170. doi: 10.5281/uh.vi286.3215 Recuperado 9 de noviembre 2025 de https://revistas.uh.cu/revuh/article/view/3215

[ref39] Gutiérrez A. B. Mansilla H. O. Clases y reproducción social: el espacio social cordobés en la primera década del siglo XXI, vol. 52; 2; 5-2015: Universidad Complutense de Madrid; Política y Sociedad, 409–444 Recuperado 9 de noviembre 2025 de: https://ri.conicet.gov.ar/handle/11336/51854.

[ref40] Hidalgo D. R. Turtós L. Caballero Á. y Martinol J. R. (2016). Relaciones interpersonales entre cuidadores informales y adultos mayores. Novedades en Población 12, 77–83. Recuperado 9 de noviembre 2025 de http://www.novpob.uh.cu/index.php/NovPob/article/view/226

[ref41] Hintze S. Y Costa M. I. (2011). “La reforma de las asignaciones familiares 2009: aproximación al proceso político de la transformación de la protección”. En C. Hintze Danani Y S. (coords.), Protecciones y desprotecciones. La seguridad-2010. Los Polvorines: UNGS.

[ref9013] Instituto de Estudios e Investigaciones sobre Trabajo (IEIT). (2004). El sistema de empleo en Cuba. MTSS: Fondo digital.

[ref9014] Jiménez Zunino C. (2011). Movilidad social intergeneracional en la Argentina reciente: Un análisis a partir de la estructura ocupacional. Sociohistórica 29, 167–196.

[ref42] Kergoat, (2010). Dinâmica e consubstancialidade das relações sociais. En: Revista Novos estudos CEBRAP, 86, março 2010, pp. 93–103

[ref43] Kessler G. Y. Espinoza V. (2003). Movilidad social y trayectorias en Buenos Aires. Rupturas y algunas paradojas. Santiago de Chile: CEPAL.

[ref44] Krause M. (2016). La interseccionalidad entre clase y género: Un acercamiento desde los relatos de vida. Lavboratorio: Revista de Estudios sobre Cambio Estructural y Desigualdad Social 27, 91–111. Recuperado 8 de agosto 2025 de https://publicaciones.sociales.uba.ar/index.php/lavboratorio/article/view/1650

[ref45] Laire C. (2016). El desarrollo en la primera infancia en Cuba la experiencia de un sistema integrado y ampliado para que todos los niños y las niñas comiencen la vida de la mejor manera. La Habana, Cuba: Unicef. Recuperado de https://www.unicef.org/cuba/informes/el-desarrollo-en-la primera-infancia-en-cuba

[ref46] Lara T. (2011). Mujeres en tránsito. La Habana, Cuba: Agencia Española para la Cooperación Internacional y el Desarrollo en Cuba.

[ref47] Lazcano A. y Colina H. (2020). Política de cuidados para la vejez: Apuntes en torno a la realidad cubana. Rebelión, s/p. Recuperado de http://progresosemanal.us/20200116/politica-de cuidados-para-la-vejez-apuntes-en-torno-a-la-realidad-cubana/ (Accessed September 11, 2025).

[ref9016] Llópiz Hernández A. F. (2008). Cuba, Estructura Social Desigualdades y Política Salarial Monografías: Recuperado de , (https://www.monografias.com/trabajos82/cuba-estructura-social-desigualdades/cuba-estructura-social-desigualdades

[ref48] Lustig N. (2017). Inequality and fiscal redistribution in Latin America: Declining but still far too high. Washington, DC: Center for Global Development.

[ref49] Mac-Clure O. Barozet E. Maturana V. (2014). Inequality, middle class and territory in Chile: global middle class or multiple mesocracy according to territories? EURE 40, 163–183. doi: 10.4067/S0250-71612015000300005

[ref9011] Martinez-Franzoni J. M. (2008). Welfare regimes in Latin America: capturing constellations of markets, families, and policies. Latin American Politics and Society 50, 67–100. Available online at: http://www.jstor.org/stable/30130857

[ref50] Martínez Franzoni J. Sánchez-Ancochea D. (2018). Undoing segmentation? Latin American health care policy during the economic boom. Soc. Policy Adm. 52, 1181–1200. doi: 10.1111/spol.12434

[ref51] Más S. (2018). En la encrucijada del cuidado. La Habana, Cuba: Editorial de la Mujer. Recuperado de http://bibliotecadegenero.redsemlac cuba.net/wp-content/uploads/2019/09/18_EdMuj_MS_ELE.pdf

[ref52] Maurizio R. Beccaria L. Monsalvo Y A. (2022), “Labour formalization and inequality: the distributive impact of labour formalization in Latin America since 2000.” Revista Development and Change, Vol. 53, No, pp. 117–165. Recuperado 9 de junio 2026 de https://econpapers.repec.org/article/bladevchg/v_3a53_3ay_3a2022_3ai_3a1_3ap_3a117.htm

[ref53] Méndez M. L. Gayo M. (2007). El perfil de un debate: Movilidad y meritocracia. Contribución al estudio de las sociedades latinoamericanas. En R. Franco . (Coords.), Estratificación y movilidad social en América Latina. Transformaciones estructurales de un cuarto de siglo (pp. 121–157). Santiago de Chile: LOM.

[ref54] Montano S. Y Calderón C. (coords.) (2010). El cuidado en acción. Entre el derecho y el trabajo. Cuadernos de la CEPAL No. 94. Santiago de Chile: CEPAL. Recuperado de https://www.cepal.org/es/publicaciones/27845 cuidado-accion-derecho-trabajo

[ref9017] Munevar D. Pineda J. (2020). Documento caso Colombia (Colección Horizontes del cuidado). Buenos Aires: Fundación Medifé.

[ref55] Muñiz Terra L. (2012). Carreras y trayectorias laborales: Una revisión crítica de las principales aproximaciones teórico-metodológicas para su abordaje. Revista Latinoamericana de Metodología de las Ciencias Sociales 2, 36–55. https://ri.conicet.gov.ar/handle/11336/75873

[ref56] Muñiz Terra L. (2018a). “Una propuesta cualitativa para la comprensión de la realidad social: El enfoque biográfico comparado” in Actas del VI Encuentro Latinoamericano de Metodología de las Ciencias Sociales* (Cuenca, Ecuador: Universidad Nacional de La Plata (UNLP)). 7–9 November

[ref57] Muñiz Terra L. (2018b). El análisis de acontecimientos biográficos y momentos bifurcativos: Una propuesta metodológica para analizar relatos de vida. Forum Qualitative Social Research 19, 1–25. doi: 10.17169/fqs-19.2.3057

[ref58] Muñiz Terra L. Ambort M. (2026). Entre el descenso y el desclasamiento en las sociedades capitalistas dependientes. Procesos de biografización en las trayectorias de movilidad en la Argentina posconvertibilidad (2003-2019). Revista Contemporánea 16, 1–23.

[ref59] Muñiz Terra L. Ambort M. (2024). Movilidad social y experiencias reflexivas: Procesos de hibridación en trayectorias de clase. Estudios Sociológicos 42:e2390. doi: 10.24201/es.2024v42.e2390

[ref60] Muñiz Terra L. (2026). “The comparative biographical perspective: an approach for the analysis of social inequalities” in Handbook of biographical research in the social sciences. eds. Rosenthal G. Becker J. Pohn-Lauggas M. Worm A. (Edward Elgar Publishing).

[ref61] Muñiz Terra L. Ambort M. E. Iucci M. (2021). Desigualdades sociales a contraluz: Un análisis a partir de trayectorias de clase en Argentina. Revista Sociedade e Cultura 24, 1–42. doi: 10.5216/SEC.V24.63396

[ref62] Muñiz Terra L. Hasicic C. Maturano Loreiro M. (2014). Carreras laborales de varones y mujeres en la industria del calzado y del petróleo en el contexto de reestructuración empresarial argentino: análisis desde una perspectiva de género. Revista Géneros 15, 57–93.

[ref63] Muñiz Terra L. Roberti M. E. Iucci M. (2026). “Welfare and care policies in Latin America: theoretical and methodological discussions and persistent challenges for comparative analysis” in Public policies and social inequalities in transformation. Comparative insights from Europe and Latinamerica. eds. Fortuntato V. Sandra Fachelli P. L. R. Y. (Milano: Franco Angeli edit).

[ref64] Muñiz Terra L. Rubilar Donoso G. (2022). Social inequalities in Argentina and Chile: a comparative analysis of welfare models, labour policies, and occupational trajectories from a biographical perspective. Journal of Politics in Latin America 14, 1–24. doi: 10.1177/1866802X221095427

[ref65] Muñiz Terra L. Verd J. M. (2021). “Social inequalities and life trajectories: theoretical–methodological elements for the comparative analysis of inequality” in Comparative analysis of social inequalities between Europe and Latin America. eds. López-Roldán P. Fachelli S. (New York: Springer), 295–330.

[ref66] Pautassi L. Faur E. y Gherardi N. (2004). Legislación laboral en seis países latinoamericanos. Avances y omisiones para una mayor equidad. Serie Mujer y Desarrollo n. 56. Santiago de Chile: CEPAL.

[ref67] Pautassi L Y Zibecchi C. (2013) “Adultos mayores, cuidado e inclusión en la agenda de la seguridad social. La transición infinita”. Grosman En C. (dir.), Los adultos mayores y la efectividad de sus derechos (cap. 3, pp. 103–145). Santa Fe: Rubinzal-Culzoni.

[ref9018] PCC (2016). Conceptualización del modelo económico y social cubano de desarrollo socialista. La Habana: Congreso del PCC.

[ref9019] PCC (2017). Documentos del 7mo. Congreso del Partido, vol. junio 2017. La Habana: Editora Política.

[ref68] Peña A. (2013). “La reproducción de la pobreza en territorios periféricos de La Habana. Lecturas desde la óptica de los regímenes de bienestar en el contexto cubano actual” in Tesis en opción al grado de Doctora en Ciencias Sociológicas (Cuba: Universidad de La Habana).

[ref69] Pla J. (2016). Condiciones objetivas y esperanzas subjetivas: Movilidad social y marcos de certidumbre. Un abordaje multidimensional de las trayectorias de clase. Buenos Aires: Autores de Argentina.

[ref70] Poy S. (2024). Informalidad y trabajadores pobres en Argentina (2003-2023). El Trimestre Económico 91, 809–845. doi: 10.20430/ete.v91i364.2543

[ref71] Proenza Z. (2013). El sistema de apoyo formal para los adultos mayores en el municipio de Camagüey. Novedades en Población, 9, pp. 24–37. Recuperado de http://www.novpob.uh.cu/index.php/NovPob/ article/view/46

[ref72] Proveyer C. Fleitas R. González R. Múnster B. Auxiliadora Y. M. (2011). “50 años después” in Mujeres en Cuba y cambio social. La Habana (Cuba: Oxfam).

[ref9020] Pujadas Muñoz J. J. (1992). El método biográfico: El uso de las historias de vida en ciencias sociales: CIS.

[ref73] Riveiro M. (2017). Apuntes críticos sobre las relaciones de género en los estudios de movilidad social intergeneracional. Labvoratorio 27, 113–129.

[ref9021] Rodríguez de la Fuente J. J. Y Sosa M. L. (2025). Desigualdad y pobreza en tiempos de Milei. Revista Sociedad, Universidad de Buenos Aires, 1–19. 10.62174/rs.10585.

[ref74] Rodríguez Enríquez C. Marzonetto G. (2015). Organización social del cuidado y desigualdad: el déficit de políticas públicas de cuidado en Argentina. Revista Perspectivas de Políticas Públicas 4, 103–134. doi: 10.18294/rppp.2015.949

[ref75] Romero M. (2012). “Las labores de cuidado infantil y el nuevo marco de regulaciones para el ejercicio del cuentapropismo en Cuba” in Revista digital Empleate 1/ 2012. Federación de Asociación de Mujeres María Laffitte (España: Sevilla).

[ref76] Romero M. (2014). La conciliación con corresponsabilidad social: algunas estrategias. Recuperado de www. (semlac.com). (Accessed August 8, 2025).

[ref78] Romero M. (2019). El trabajo doméstico a domicilio remunerado en Cuba. Un estudio de caso en Miramar. Anales de la Academia de Ciencias de Cuba, 9, 127–129.

[ref79] Romero M. (2020) Tendencias de los estudios sobre cuidados en Cuba (2000 – 2020). En Alfonso G. Romero M. Echevarría D. Proveyer C. Lara T. Los cuidados en la ruta hacia la equidad en Cuba. Disponible en: https://biblioteca.clacso.edu.ar/Cuba/if-mctma/20210330014636/Los-cuidados-ruta.pdf

[ref9024] Romero Almodóvar M. (2010). Domésticas y Revolución en Cuba: entre cambios y desafíos. La Habana: Universidad de La Habana, Departamento de Sociología.

[ref80] Roque Yamila Azcuy Lucrines Y Toledo Leticia (2015). Política social para la atención a la vejez: una necesidad para Villa Clara. Novedades en Población, (11)22, pp. 20–28. Recuperado de http://www.novpob.uh.cu/ index.php/NovPob/article/view/300/331

[ref9022] Salvador Soledad (2011). Hacia un sistema nacional de cuidados en el Uruguay. En: Rico Maria Nieves (coordinadora). El desafío de un sistema nacional de cuidados para el Uruguay. Serie Seminarios y Conferencias. División Desarrollo Social, CEPAL. Recuperado de . (https://repositorio.cepal.org/server/api/core/bitstreams/3585eded-e350-4165-b5db-c703cc606b07/content)

[ref81] Salvia A. (2013). Heterogeneidad estructural y desigualdad social en la Argentina de las últimas dos décadas de historia económica. Revista de Investigación en Ciencias Sociales, núm. 84, 46–55.

[ref82] Solís P. Boado M. (2016). And yet it moves… social stratification and intergenerational class mobility in Latin America. Mexico City: Centro de Estudios Espinosa Yglesias, El Colegio de México.

[ref83] Southwell M. (2011). Política educativa en la Argentina reciente: entre la ampliación de derechos y las tensiones de la implementación. en Propuesta Educativa 20, 25–34.

[ref84] Trujillo L. (2017). La Argentina kirchnerista: Alcances y límites de una experiencia democrática sobre la distribución del ingreso (2003-2015). Polis. Santiago de Chile 16, 99–126.

[ref9023] Vera J. Salvia A. Bonfiglio J. I. Giannecchini A. (2025). Ajuste libertario, crisis y estabilización: Efectos sobre la dinámica de la pobreza y la desigualdad social. Revista de Políticas Sociales Urbanas: Ciudadanías https://revistas.untref.edu.ar/index.php/ciudadanias/article/view/2415.

[ref85] Wainer Andrés (2018). Economía y política en la Argentina kirchnerista (2003-2015). Revista Mexicana de Sociología. Ciudad de México, v. 80, n., pp. 323–351.

[ref86] Zabala M. D. (2010). Familia y pobreza en Cuba: estudio de casos. La Habana: Publicaciones. La Habana: Acuario / Centro Félix Varela.

[ref87] Zabala M. D. Echevarría D. (2020). Las políticas sociales para la Cuba del 2030: elementos para su diseño e implementación. Revista Economía y Desarrollo, 164, 1–13.

[ref88] Zabala M. D. Echevarría D. (2025). Políticas sociales en Cuba 2019-2024: apuntes para una reforma. En Diaz I. Sosa N., Miradas a la Economía Cubana: apuntes para una reforma (págs. 127–136). La Habana: Fundación Friedrich Ebert.

