# Supplementary material for: Between work and care: a comparative study of the trajectories of women from different social classes in Cuba and Argentina
Source: Front Sociol. 2026 Apr 22;11:1756504. doi: 10.3389/fsoc.2026.1756504 (PMC13148266; doi:10.3389/fsoc.2026.1756504)
Supplement: Supplementary file 1 [file Data_Sheet_1.docx]

**APPENDIX 1. INTERVIEW GUIDE**

**PRODUCTIVE WORK AXIS**

| **Dimension** | **Definition** | **Questions** |
| --- | --- | --- |
| Summary of work history | Briefly reconstruct the interviewee's occupational sequence | The idea is to recover your work history. To begin, I would like you to tell me about your work life. How many jobs have you had in your life, and what were they?  APPLY WORK BIOGRAPHICAL GRID. |
| **SECTION A: FIRST JOB** | | |
| Characterization of first work | Selection of the first job, based on the criteria used by the interviewee.  Characterize it based on its temporality, form of access, motivations, tasks, conditions of  hiring | What was your first job? How old were you? How did you get it? Did any family members, friends, or acquaintances help you find it? What tasks did you perform? During  How long did you work on it? What motivated you to start working on it at that time?  What were the terms of employment? Before that job, did you do any unpaid informal work or help a family member in their business? |
| Inheritance and social capital | The idea is to observe the interfamily and social transmission of social capital. | How did you get this job? Did a family member, friend, or acquaintance help you find it? Where does that person work?/Where do you know them from? Did they require any prior knowledge to get this job? What was it? What tasks did you perform?  Where did you learn to do them? Did those tasks change over time? |
| Evaluations of the first job | Evaluations of the first job based on enjoyment of the task, learning, relationships  with later work | What did you like most and least about the tasks you performed?  Why? Do you feel you learned something? What? (a trade, an administrative skill, etc.) Who taught you how to do that? Were you able to apply it in subsequent jobs? |
| Termination | Identify the method of leaving the first job  most important | Why did you stop working on that? How did you feel about this situation? |
| **SECTION B. TWO MOST IMPORTANT JOBS** | | |
| Important works  TWO MORE JOBSIMPORTANT | Identify the two most relevant jobs according to which one has been longer, that is, the one in which you have remained for the longest time.  If any of the listed jobs is the first or current job, inquire about the third most relevant job. | Without considering your current job, what were the two most important jobs in your working life where you stayed the longest? |

|  | If you only held one job your entire life, skip to the section. |  |
| --- | --- | --- |
| First major job | | |
| First most important task: characterization | Characterize it based on its temporality, form of access, hiring conditions, knowledge,  tasks | What year did you start this job? How old were you when you started and when you left this job? How many hours a day did you work? Where did you work (physical location)? How many days a week? Did you have a contract? Was it a stable job with social security, or was it informal? Were you an employee or self-employed? What did the establishment do?  How did you get it? Did any family member, friend, or acquaintance help you find this job? Did they require any prior knowledge to get the job? What was it? What tasks did you perform? How many employees were there? |
| Career path | Identify if there was a possibility of career advancement within the first most important job | Did you receive any promotions or better job opportunities there? If no: Why? If yes: How did you achieve the promotion or change of duties/position? What did it entail in terms of tasks and benefits (financial, prestige, etc.)? Did you have staff/employees reporting to you? How many?  Why do you think you got to that point? |
| Inheritance and social capital | See the interfamily and social transmission of social capital. | How did you get this job? Did a family member, friend, or acquaintance help you find it? Where does that person work?/Where do you know them from? Did they require any prior knowledge to get this job? What was it? What tasks did you perform? Where did you learn to perform them? Did those tasks change over time? |
| Termination | Identify the method of leaving the first most important job | Why did you stop working on that? How did you feel about this situation? |
| Evaluations of the first best work | Evaluations of the first best work based on enjoyment of the task, expectations, learning, links to previous work  later | Did you enjoy the tasks you performed? Why? Did it meet your expectations when you started working there? In what way? Do you feel you learned anything? What? (a trade, an administrative skill, etc.) Were you able to apply it in subsequent jobs? |
| Second most important job: | | |
| Second most important job:  characterization | Characterize it based on its temporality, form of access, hiring conditions, knowledge, tasks | What year did you start this job? How old were you when you started and when you left this job? How many hours a day did you work? How many days a week? Did you have a contract? Was it a stable job with social security, or was it informal? What did the establishment do? |

|  |  | How did you get it? Did a family member, friend, or acquaintance help you find this job? Did they require any prior knowledge to get the job?  Which one(s)? What tasks did you perform? |
| --- | --- | --- |
| Career path | Identify if there was a career opportunity within the second most important job  important | Did you get a promotion or better job opportunities there?In case of a negative response:Because?If the answer is yes:How did you achieve the promotion or change in duties/positions? What did it entail in terms of tasks and benefits (financial, prestige, etc.)? Did you end up having staff/employees under your supervision? Why do you think you reached that point? |
| Inheritance and social capital | See the interfamily and social transmission of social capital. | How did you get this job? Did a family member, friend, or acquaintance help you find it? Where does that person work?/Where do you know them from? Did they require any prior knowledge to get this job? What was it? What tasks did you perform?  Where did you learn to do them? Did those tasks change over time? |
| Termination | Identify the method of leaving the second most important job | Why did you stop working on that? How did you feel about this situation? |
| Evaluation of the second best work | Evaluations of the second best work based on enjoyment of the task, expectations, learning, links to previous work  later | Did you enjoy the tasks you performed? Why? Did it meet your expectations when you started working there? In what way? Do you feel you learned anything? What? (a trade, an administrative skill, etc.) Were you able to apply it in subsequent jobs? |
| **SECTION C: CURRENT WORK AND PROJECTIONS** | | |
| Current job:  characterization | Characterize it based on its temporality, form of access, hiring conditions, knowledge, tasks | Where do you currently work? How long have you worked there? How did you get the job?  How many hours do you work? How many days a week? Do you have a contract? Is it a stable job with social security or is it informal? What kind of business does the establishment do?  How did you get this job? Did a family member, friend, or acquaintance help you find it? Did they require any prior knowledge to get the job? What was it? What tasks did you perform? Where did you learn to perform them? Did those tasks change over time? |
| Evaluation of current work | Evaluations of current work based on enjoyment of the task, expectations,  learning | Did you enjoy the tasks you performed? Why? Do you think it met the expectations you had when you started working in this field? In what way? Do you feel you learned anything? What? (a trade, an administrative skill, etc.) |
| **Inheritance and social capital** | See the interfamily and social transmission of social capital. | How did you get this job? Did a family member, friend, or acquaintance help you find it? Where does that person work? / Where do you know them from? To get that job |

|  |  | Did they require you to have any prior knowledge? What was it? What tasks did you perform? Where did you learn to perform them? Did those tasks change over time? |
| --- | --- | --- |
| Recognition | Recognition based on revenue, valuations | Do you receive an income for your work? Do you feel it's sufficient for the work you do? Aside from income, do you feel your work is adequately valued?  Why? Are you interested in the work you're currently doing? What positive and negative aspects do you find in your current job? |
| Future projection | Future projection | Do you plan to work in the same field you currently work in in the future?  Because?If no:What kind of work would you like to do? Why? What conditions do you think would be necessary to get a job like that? Have you done any job searches lately?  How do you imagine yourself in 10 years? (To support the imagery in this question, you can also use age (if you are 35, for example, ask how do you imagine yourself at 45?) What would you like to be doing? What would you never like to work as? Why? |
| **SECTION D: GENERAL QUESTIONS ABOUT THE CAREER PATH:** | | |
| Periods of unemployment or inactivity | Identify the periods in the career path where one did not work, reasons, reintegration | Have you ever been unemployed or inactive due to maternity, studies, or caregiving?  When? Why? Did you find a job quickly?If no:Why do you think you couldn't find a job at that time? How did you manage to earn an income during that period? How did you re-enter the job market? |
| Moonlighting | Identify the moments in the career where he/she held more than one job at the same time  time, and the reasons | At what points in your career did you have more than one job at the same time?  What were the reasons? |
| Institutional support | Identify if there was any relevant institutional support in the development of the  work history | Do you consider that any public or private institution (educational, company, NGO, foundation,  Did any program (study grants, scholarships, aid, job placement programs or training) significantly contribute to the development of your career path? |
| Intergenerational mobility | Social origin | Who did you live with during your childhood and adolescence? Let's think, for example, when you were 15 years old, what was your household like? What did the older people in that household do for a living? (Identify their relationship to you, their occupation, and the tasks they performed in their jobs.)  employment relationship, working hours)? Who could be identified as the main breadwinner of the household in economic terms? Did you help with the work of your father/mother/whoever is responsible according to the household composition? |
|  | Comparison with parents' trajectory | What similarities and differences do you see between your career path and that of your parents? Do you think your job is better or worse than theirs? In what way? |

**CARE AXIS/UNPAID WORK**

| **DIMENSION ASPECT** | **POSSIBLE QUESTIONS** | | |
| --- | --- | --- | --- |
| Family composition | Could you briefly tell me at what age you left your family home? When was your current home formed? What is your family like? | | |
|  |  | APPLY CARE BIOGRAPHY. |  |
| Current caregiving tasks | Could you briefly tell me about your childcare and eldercare responsibilities that you usually perform during the week?  Who are they (relationship relationship)? What types of dependency or care needs do they have? What are their ages? | | |
| Task and time allocation | How do you manage your work schedule with caregiving responsibilities? Could you describe a typical workday?  How are caregiving tasks distributed? | | |
| Birth and raising of children | Do you have children? Regardless of whether your children are grown, could you tell me if you worked for pay during your pregnancies?  Did you have social security coverage? For how long? Etc.  How did you organize yourself during pregnancy and after the birth of each of your children? Did you have access to maternity leave? For how long? | | |
| Care for the elderly | Do you have to care for an elderly family member? What tasks do you perform? Do you do it alone, or does another family member also help? | | |
| Reconciliation of productive and reproductive work | Have caregiving responsibilities had any impact on your work life? If so, how would you rate that impact?    At some point in your working life, did you have to leave your job due to caregiving responsibilities? Explore whether this decision was due to a lack of available services, distrust in their quality, or insufficient income to maintain such services. Delve deeper into the role of the employer/institution in relation to caregiving.    Did you have to turn down any job, promotion, or career advancement opportunities because of your caregiving responsibilities?    Have you received any financial or material compensation for your care work? | | |
|  | How do you value the cost of care work in your professional life? What contributions has your care work made to your career path? | | |

**APPENDIX 2. Work Grid**

Interviewee ID:

**Profile as shown:**

**Year of Birth:**

**Place of birth:**

**Current place of residence:**

**Ethnic-racial ancestry:**

| **Life lived** | | | | | | | | **Changes in trajectory** | | **External dimensions** | | |
| --- | --- | --- | --- | --- | --- | --- | --- | --- | --- | --- | --- | --- |
| **Life lived – Stages and states** | | | | | | | | **Transitions** | | **Institutional resources (meso)** | | **Macrosocial** |
| Age | Year | Stage (work no. and migration?) | Type of work. Employment status  in main work | Multiple employment | Required training | Type of occupation | Related intentions and preferences | Family or personal events (births, marriages, illnesses) | Significant external events | Public Resources (institutional intervention) | Private resources (institutional intervention) | Type of Welfare State or Employment Model |
|  |  |  |  |  |  |  |  |  |  |  |  |  |
|  |  |  |  |  |  |  |  |  |  |  |  |  |
|  |  |  |  |  |  |  |  |  |  |  |  |  |

**APPENDIX 3. Grid on Care Work**

Interviewee ID:

**Profile as shown:**

**Year of Birth:**

**Place of birth:**

**Current place of residence:**

**Ethnic-racial ancestry:**

| **Life lived – Stages and states** | | | | | | **Transitions** | | **Expectations** | **Institutional resources (meso)** | **Macro-context** |
| --- | --- | --- | --- | --- | --- | --- | --- | --- | --- | --- |
| **Age** | **Year** | **Household composition**  **(Stage)** | **Dependents**  (children, senior citizens, people with disabilities)  Also consider dependents outside the home | **Care work management***  (burden on interviewee, other participants, care centers, purchase of care) | **Domestic work management****  (burden on interviewee, other participants, outsourcing and purchase of domestic labor) | **Births, deaths, marriages, common-law unions, divorces, separations** | **Moving / Migration** | **Expectations**associated with transitions and changes of state | **Reception of social policies related to care or family support, public care centers, etc.** | **Changes in welfare regimes, economic crises, policies and public programs** |
|  |  |  |  |  |  |  |  |  |  |  |
|  |  |  |  |  |  |  |  |  |  |  |
|  |  |  |  | . |  |  |  |  |  |  |

* Illustration of care work: feeding, dressing, bathing, assisting with transfers and daily living, coordinating or monitoring medical appointments, and providing care in case of illness. In the specific case of children: helping them with homework, attending educational events, putting them to bed, and dressing them.

** Illustration of domestic work: daily cleaning of the home (sweeping, washing dishes, washing clothes), buying and preparing food, managing the payment of household bills, managing social activities.
